# Ecological function of key volatiles in *Vitex negundo* infested by *Aphis gossypii*

**DOI:** 10.3389/fpls.2022.1090559

**Published:** 2023-01-12

**Authors:** Qingxuan Xu, Changbing Wu, Da Xiao, Zhenyu Jin, Changrong Zhang, Séverin Hatt, Xiaojun Guo, Su Wang

**Affiliations:** ^1^ Institute of Plant Protection, Beijing Academy of Agriculture and Forestry Sciences, Beijing, China; ^2^ Hubei Engineering Research Center for Pest Forewarning and Management, College of Agriculture, Yangtze University, Jingzhou, China; ^3^ Institute of Plant Protection, Guizhou Academy of Agriculture Sciences, Guiyang, Guizhou, China; ^4^ Agroecology and Organic Farming, Institute of Crop Science and Resource Conservation, University of Bonn, Bonn, Germany

**Keywords:** HIPVs, indigenous plants, *Harmonia axyridis*, chemical ecology, woody plants

## Abstract

Herbivore induced plant volatiles (HIPVs) are key components of plant-herbivorous-natural enemies communications. Indeed, plants respond to herbivores feeding by releasing HIPVs to attract natural enemies. The present study analyses the effect of HIPVs of *Vitex negundo* (Lamiaceae), an indigenous plant species in northern China, on the predatory ladybug species *Harmonia axyridis.* Y-tube olfactometer bioassay showed that *H. axyridis* adults were significantly attracted by *V. negundo* infested by the aphid *Aphis gossypii.* We analyzed and compared volatile profiles between healthy and *A. gossypii* infested *V. negundo*, screened out the candidate active HIPVs mediated by *A. gossypii* which could attract *H. axyridis*, and tested the olfactory behavior of the candidate active compounds on *H. axyridis*. The gas chromatography-mass spectrometry analysis showed that five volatile compounds were significantly up-regulated after *V. negundo* infestation by *A. gossypii*, and five substances were significantly down-regulated in the terpenoid biosynthesis pathway. The olfactory behavior response showed that *H. axyridis* has significant preference for sclareol, eucalyptol, nonanal and α-terpineol, indicating that this chemical compounds are the important volatiles released by *V. negundo* to attract *H. axyridis*. This study preliminarily clarified that *V. negundo* release HIPVs to attract natural enemies when infected by herbivorous insects. The description of the volatile emission profile enriches the theoretical system of insect-induced volatile-mediated plant defense function of woody plants. Applications in crop protection would lie in designing original strategies to naturally control aphids in orchards.

## Introduction

Conservation biological control (CBC) takes full advantage of the surrounding environment of the target area to conserve natural enemy insects and balance the ecological relationship between natural enemies and pests (Tooker et al., 2020). How to effectively use ecological factors including landscape diversity, functional plants, and volatile organic compounds (VOCs) to improve the efficiency of natural enemy insects in biological control of pests is hotspot in CBC programs (Turlings and Ton, 2006; Gurr et al., 2017; Hatt et al., 2019). In this context, the information substance that links the communication between plants and insects, i.e. herbivore induced plant volatiles (HIPVs), have received more attention over the recent years (Li and Blande, 2017; Turlings and Erb, 2018).

As chemical signals between plants and insects, HIPVs can improve the defense ability of plant and mediate the interaction between plant and insect community to affect the behavior of insects (Song et al., 2017). HIPVs are mainly divided into terpenoids, green leaf volatiles (GLVs), nitrogen- and sulfur containing compounds (Aartsma et al., 2017; Ye et al., 2019). Terpenoids are the most abundant plant volatiles and the most common compounds induced by pests (Dicke, 2009). Among them, volatile substances such as monoterpenes and diterpenes are released after pest infection and directly participate in plant defense by attracting natural enemies to avoid further damage (Dudareva et al., 2004; Cheng et al., 2007). Studying the biosynthetic pathways of these volatile compounds helps to explore their effects on plant biological characteristics (Cagliero et al., 2020). For instance, terpenoids are released from the leaves of the hybrid *Populus trichocarpa* Torr. & A.Gray and *Populus deltoides* W.Bartram (Salicaceae) when infested by *Phyllobius piri* Linnaeus (Coleopotera: Curculionidae) (Blande et al., 2007) and terpenoids, such as (*E*)-4,8-dimethyl-1,3,7-nonatriene and 4,8,12-trimethyl-1,3,7,11-tridecatetraene, can induct defense-related genes (Arimura et al., 2001). Hence, terpenoids play an important role in tritrophic interactions and in the direct and indirect defense of plants.

HIPVs-mediated plant-insect interactions have been extensively studied in herbaceous or gramineous plants (Heil, 2014; Turlings and Erb, 2018). Findings led to designing and managing diversified cropping systems using selected functional plants and releasing selected chemical compounds known to repel insect pests and to attract their natural enemies (Khan et al., 2008; Xu et al., 2018a). In contrasts, the role of HIPV produced by woody plants in attracting pest predators in the vicinity of orchard ecosystems have been rarely considered to our knowledge.

*Vitex negundo* L. var. *heterophylla* (Franch.) Rehd (Lamiaceae) is a perennial shrub indigenous in northern China. It is an important nectar plant widely distributed in semi-natural habitats (Gill et al., 2018), notably in orchard surroundings. At present, few ecological studies explored its potential benefits as a non-crop plant supporting natural enemies of pests. Previously, it was showed that *V. negundo* infested by the aphid *Aphis gossypii* Glover (Hemiptera: Aphididae) significantly attracts lacewings *Chrysopa formosa* Brauer (Neuroptera: Chrysopidae) (Chen and Feng, 2014). While in their study, Chen and Feng (2014) did not identify the mechanism explaining the attraction of aphid predators to *V. negundo*, we hypothesized that volatiles released by the *V. negundo* plants mediated by *A. gossypii* are used as chemical information structuring the tri-trophic interactions between *V. negundo*, *A. gossypii* and predators. In this study, we first conducted field observations and highlighted that ladybugs were especially abundant on *V. negundo*. We then tested the olfactory behaviors of adults of the ladybug species *Harmonia axyridis* Pallas (Coleoptera: Coccinellidae) to *V. negundo* plants, analyzed the HIPV compounds mediated by the aphids *A. gossypii*, and lastly tested the olfactory behavioral responses of *H. axyridis* to the identified active compounds.

## Materials and methods

### Field investigation of *Vitex negundo* and peach trees in field

The occurrence dynamics of predatory natural enemies on peach trees and *V. negundo* were investigated in Changping district experimental station in Beijing, China in 2020 (116°2’ E, 40°10’ N). *Vitex negundo* plants were naturally growing in the direct surrounding of the investigated peach orchard. Every seven days from early May to early August (14 times in total), about two-year old *V. negundo* plants were surveyed in plots containing each 25 plants (in a 5 × 5 plant layout with 0.6 m between plants). Three plots were surveyed (i.e., 75 *V. negundo* plants in total) and 10 branches per plant were investigated. Larvae and adults (but neither eggs nor pupae) of ladybugs were recorded. *H. axyridis* was the most abundant species. Investigations of other habitats plants (*Artemisia sieversiana*, *Cosmos bipinnatus*, *Helianthus annuus*, *Vigna unguiculata*, *Zea mays* and *Anemarrhena asphodeloides*) performed as are described above. To investigate the presence of predators on peach trees, five points were selected at equidistance (i.e., 1.2 m between each point) through the diagonal of the peach orchard. Two peach trees were selected at each point, and 10 branches were selected on each peach tree to record the number of predatory ladybugs.

### Laboratory test set up

#### Plant materials

*Vitex negundo* seeds were collected at the experimental station in Changping district, Beijing, China, and planted in a greenhouse (25 ± 2°C, natural light) at a density of four seeds per pot (1 gallon pot). Vermiculite, perlite, peat (Pindstrup), mixed at a ratio of 1:1:4, were used as substrate, and each pot received 1 L of water every week. When *V. negundo* plants reached 10 leaves, plants of similar size were selected for the experiment.

#### Insects rearing

*Harmonia axyridis* and *A. gossypii* used in the experiments came from the laboratory populations maintained at the Institute of Plant Protection, Beijing Academy of Agriculture and Forestry Sciences. *H. axyridis* were reared in 30 cm × 30 cm × 50 cm cages and fed with *Megoura crassicauda* Mordvilko (Hemiptera: Aphididae) on *Vicia faba* L. (Fabaceae). After multiple generations of indoor reproduction, the newly emerged adults of *H. axyridis* were selected for subsequent experiments. *A. gossypii* were reared on *Cucumis sativus* L. (Cucurbitaceae). All insects were reared in climate chambers (Sanyo, MH351) at 26 ± 1°C, relative humidity of 45% ± 5%, photoperiod of 16L: 8D, light intensity of 800 lx.

#### Olfactory choice test to different treatment plants with Y-tube olfactometer

The selection preference behavior of adult *H. axyridis* to different treated *V. negundo* plants was tested by Y-tube olfactometer in insect behavior observation box. Three treatments were compared two-by-two: (i) *V. negundo* previously infested by *A. gossypii* aphids, (ii) healthy *V. negundo* plants, and (iii) a blank treatment as control. To prepare the aphid infested plants, *A. gossypii* (wingless aphids) were introduced on the leaves of *V. negundo* with a small brush at a density of 200 per pot, and the treated plants were covered with gauze. After 24 h, *A. gossypii* and their molting and honeydew were gently brushed off. The common arm of the Y-tube olfactometer was 15 cm, and the two tube arms were 10 cm. Air was introduced into the activated carbon tube by the atmospheric sampler. After passing through a long neck distillation bottle containing distilled water, the air entered the olfactometer through the rubber tube, and the airflow velocity was set to 400 mL/min. Before the experiment, adults of *H. axyridis* were starved for 24 h. Then, a single adult individual of *H. axyridis* was placed on the Y-tube main arm to observe its behavioral response. Timing started when the ladybug reached the center of the common arm tube. A choice was recorded when the ladybug crawled over the half of one of the two choice tubes and stayed in this area for more than 5 s. If no choice was made after 5 min, it was recorded as an absence of response and the individual was excluded. In the experiment, each ladybug was tested only once. After each tested ladybug, the position of the two treatments on the olfactometer arms was switched. After every five tested ladybugs, the Y-tube olfactometer was washed with alcohol and replaced by a clean olfactometer. In total, 60 male and 60 female adults were tested in each treatment comparison.

#### Analysis of *Vitex negundo* volatile compounds

Similarly than in the behavioral tests, infested plants were prepared by depositing *A. gossypii* aphids on *V. negundo* leaves at a density of 200 individuals per pot (four plants per pot) and brushing them off along with their molting and honeydew after 24h. After the treatment was completed, fresh leaves were collected from each group of *V. negundo* plants and put into plastic bags and quickly placed in liquid nitrogen. After grinding, the vortex was mixed evenly. 1 g (1 mL) of the powder was transferred immediately to a 20 mL head-space vial (Agilent, Palo Alto, CA, USA), containing NaCl saturated solution, to inhibit any enzyme reaction. The vials were sealed using crimp-top caps with TFE-silicone headspace septa (Agilent). At the time of SPME analysis, each vial was placed in 100°C for 5 min, then a 120 µm polydimethylsilioxan fibre (Agilent) was exposed to the headspace of the sample for 15 min at 100°C. After sampling, desorption of the VOCs from the fiber coating was carried out in the injection part of the GC apparatus (Model 8890; Agilent) at 250°C for 5 min in the splitless mode. The identification and quantification of VOCs was carried out using an Agilent Model 8890 GC and a 5977B mass spectrometer (Agilent), equipped with a 30 mm x 0.25 mm x 0.25 μm DB-5MS (5% phenyl-polymethylsiloxane) capillary column. Helium was used as the carrier gas at a linear velocity of 1.2 mL/min. The injector temperature was kept at 250°C and the detector at 280°C. The oven temperature was programmed from 40°C (3.5 min), increasing at 10°C/min to 100°C, at 7°C/min to 180°C, at 25°C/min to 280°C, hold for 5 min. Mass spectra was recorded in electron impact (EI) ionization mode at 70 eV. The quadrupole mass detector, ion source and transfer line temperatures were set, respectively, at 150, 230 and 280°C. Mass spectra was scanned in the range m/z 50-500 amu at 1 s intervals. Identification of volatile compounds was achieved by comparing the mass spectra with the data system library (NIST2.0) and linear retention index. Volatiles were tentatively identified with spectra and high-probability matches (> 85%) according to NIST mass spectral database. Each group was repeated three times.

#### Olfactory choice test to key volatile organic compounds with Y-Tube olfactometer

Choice tests using Y-tube olfactometers were conducted to observe the olfactory behavioral responses of *H. axyridis* to different concentrations of compounds which release was found to change significantly after *A. gossypii* infection (**Table 1**). The whole protocol was similar than when using entire leaves (see above), but with chemical compounds instead. Each compound was prepared in four concentrations, 1 μL/mL, 10 μL/mL, 100 μL/mL, 500 μL/mL (solid solute was μg/mL), and liquid paraffin was used as solvent. In the experimental treatment, 10 μL solution of the tested compound was added to a rectangular filter paper (2 cm × 1 cm) and introduced into the flavor source bottle. 10 μL liquid paraffin was added to a filter paper in the other odor bottle as a control. The filter paper was changed every hour. The calculation formula of the selection rate is as follows:


$$\text{Selection rate of A arm}=\frac{\text{Select the number of adults of A}}{\text{Total number of effective selected adults}}$$

$$\text{Selection rate of B arm}=\frac{\text{Select the number of adults of B}}{\text{Total number of effective selected adults}}$$

**Table 1:** Test compounds and their sources.

Formula	Compounds	CAS	Company	Purity
C_8_H_10_O	2-Phenylethanol	60-12-8	Macklin	99%
C_9_H_18_O	Nonanal	124-19-6	Macklin	96%
C_10_H_18_O	Eucalyptol	470-82-6	Macklin	99%
C_10_H_18_O	α-Terpineol	10482-56-1	Macklin	98%
C_15_H_24_	Valencene	4630-07-3	Macklin	75%
C_15_H_24_	(+)-Δ-Cadinene	483-76-1	Shanghai Yuanye Bio-Technology Co., Ltd.	95%
C_20_H_36_O_2_	Sclareol	515-03-7	Macklin	98%
C_25_H_43_NO_3_	Paraffin liquid	8042-47-5	Macklin	99%

“(+)” “Δ” indicates common symbols used to determine the spatial structure.

#### Statistical analyses

We marked the non-selected *H. axyridis* as ineffective selection, and calculated the ratio of effective selection of *H. axyridis* in treatment or control as selection rate. Olfactory selection results were weighted, and the effect of treatments was analyzed using a non-parametric chi-square test. Difference in volatile chemical composition between infested and non-infested plants was analyzed through an Orthogonal Partial Least Squares Discriminant Analysis (OPLS-DA) performed on log2-transformed data followed by mean centering (Anal function Metabo Analyst R package OPLSR, R Core Team, 2020). Significantly regulated metabolites between groups were determined by VIP ≥ 1 and absolute fold change FC ≥ 1. VIP values were extracted from OPLS-DA results, which also contain score plots and permutation plots, generated using R package Metabo Analyst R. In order to avoid overfitting, a permutation test (200 permutations) was performed. Identified metabolites were annotated using KEGG Compound database (http://www.kegg.jp/kegg/compound/), annotated metabolites were then mapped to KEGG Pathway database (http://www.kegg.jp/kegg/pathway.html). Pathways with significantly regulated metabolites mapped were then fed into MSEA (metabolite sets enrichment analysis), and their significance was determined by hypergeometric test’s p-values.

## Results

### Field investigation

**Figure 1:**
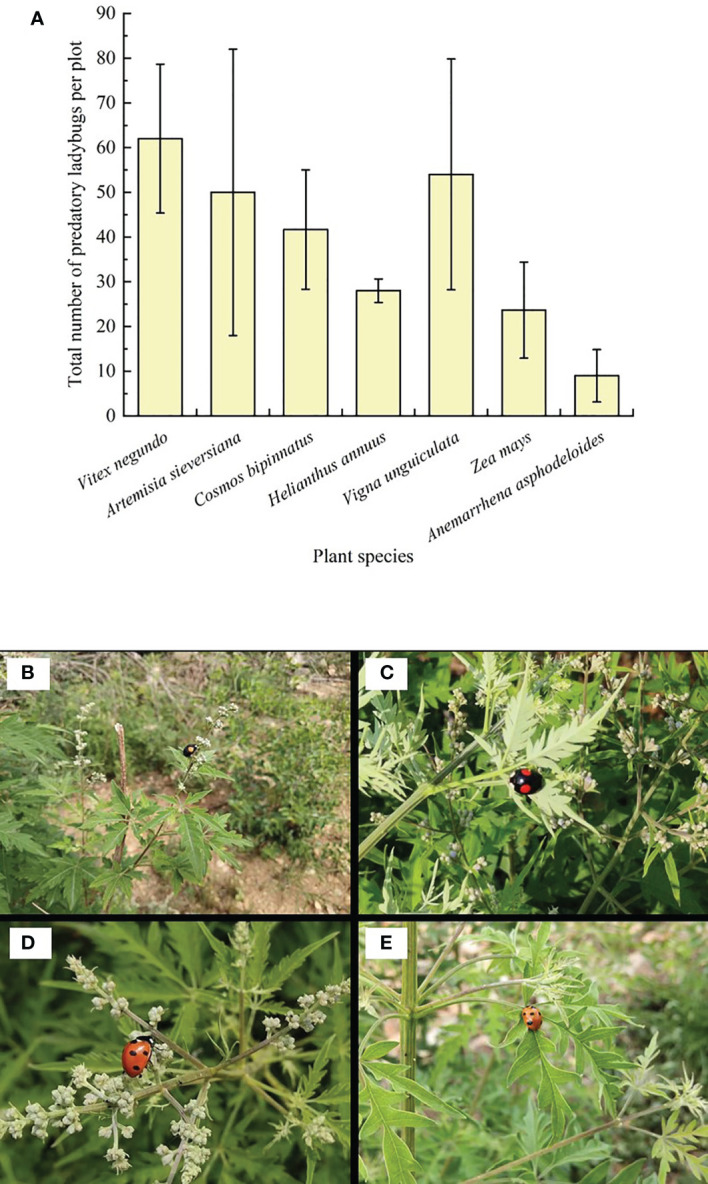
Abundance of predatory ladybugs on habitat plants and predatory ladybugs on *Vitex negundo*. Mean (± SE) per plot **(A)**, *Harmonia axyridis* **(B, C)** and *Coccinella septempunctata* **(D, E)** adults are visiting the flowers and leaves.

Predatory ladybugs were the most abundant on *V. negundo* compared to the other plant species (**Figure 1A**), showing the superior ability of *V. negundo*, compared to the other plant species in this environment, to conserve natural enemies. During the observations, it was noticed that ladybugs colonized *V. negundo* after the plants were infested by the aphid *A. gossypii*. While *A. gossypii* also infested some other plant species (e.g. *Anemarrhena asphodeloides*), the high abundance of ladybugs on *V. negundo* suggested a specific interaction between *V. negundo*, aphids and ladybugs (**Figure 1B–E**).

**Figure 2:**
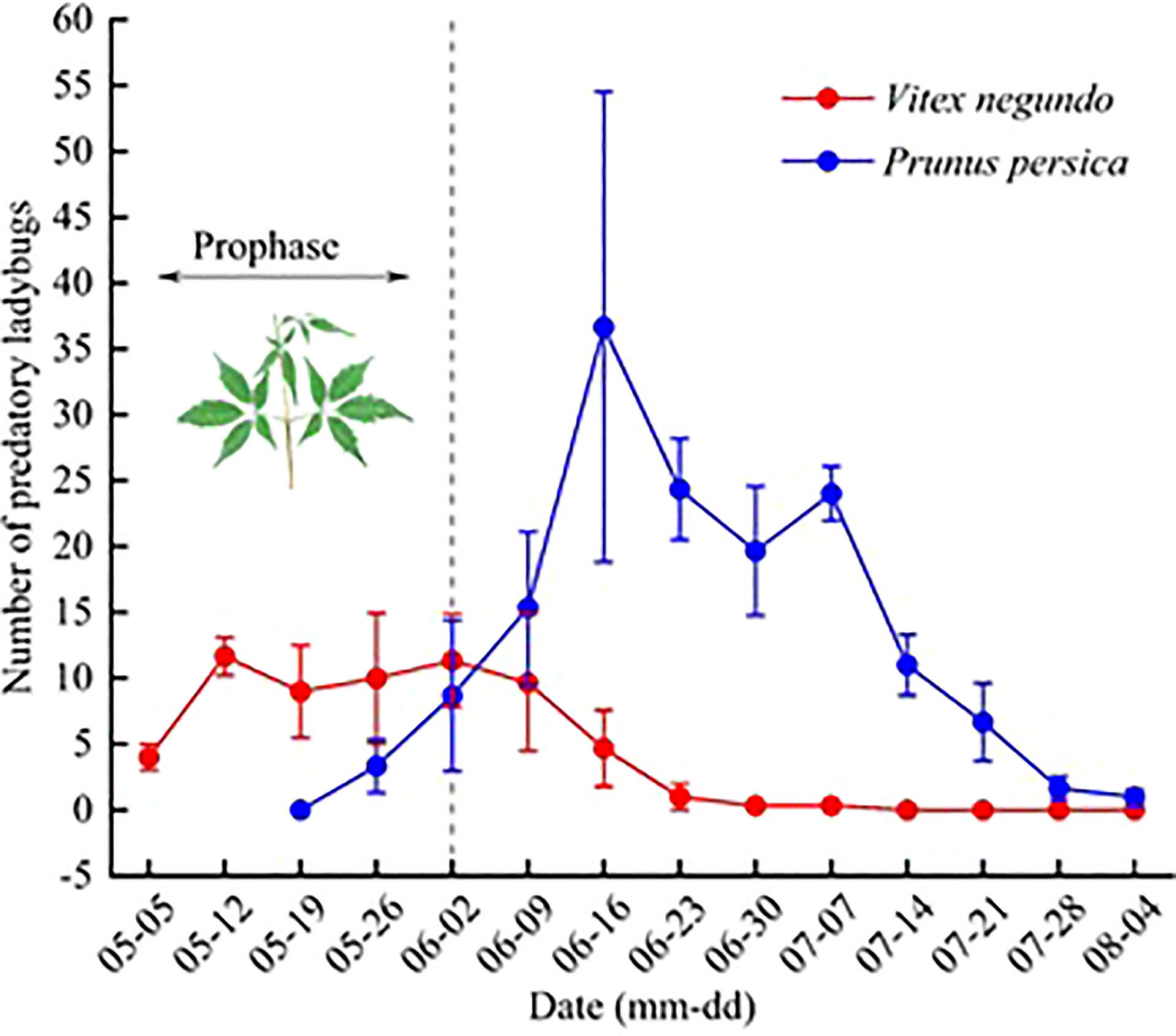
Occurrence dynamics of predatory ladybugs on *Vitex negundo* and adjacent peach trees. Note: Mean (± SE) per plot.

Dynamic results based on the occurrence of predatory ladybugs on *V. negundo* and peach trees indicate that during the prophase, from early May to early June, the ladybugs concentrated on *V. negundo*. After the beginning of June, the number of ladybugs on *V. negundo* decreased, while the number of predatory ladybugs in the peach orchard increased rapidly (**Figure 2**).

### Behavioral responses of *Harmonia axyridis* adults to plant odors

**Figure 3:**
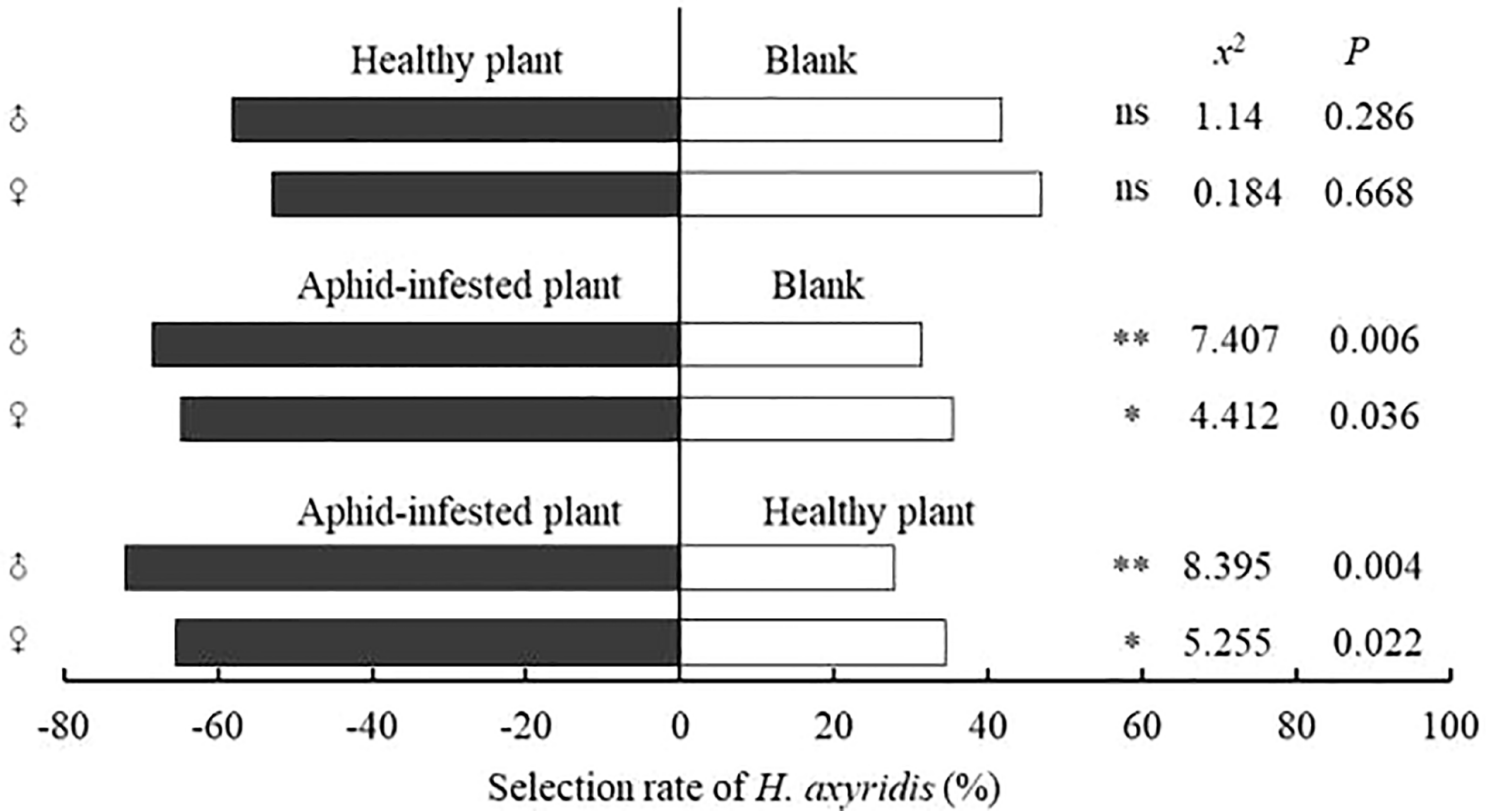
Behavioral responses of females and males of *H. axyridis* adults to different combinations in the Y-tube olfactometer assays. “*” denotes a significant difference at the *P*< 0.05 level, “**” means *P*< 0.01; “ns” indicates no significant difference.

**Figure 4:**
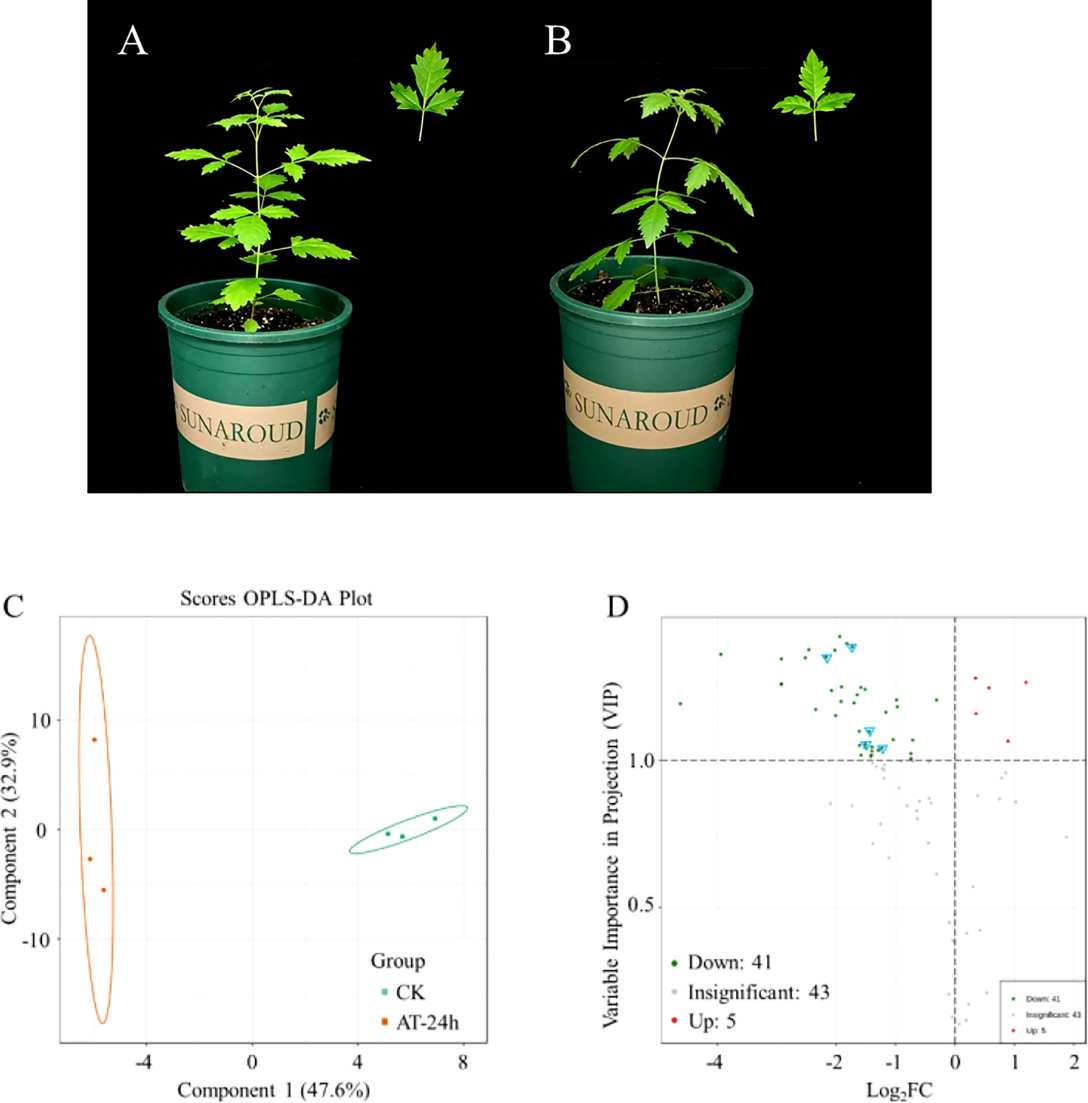
Orthogonal partial least squares-discriminant analysis (OPLS-DA) and differential material analysis of *V. negundo* plant volatile compounds. **(A, B)** *Vitex negundo* plants uninfected, and infected by *A*. *gossypii* during 24h. **(C)** Each point represents a sample, the samples of the same group are represented by the same color, with grouping using 95% confidence interval. **(D)** Each point represents a metabolite. When both VIP≥1 and FC≥1 double screening conditions are met, it is considered as a significantly up-regulated substance. The red point represents an up-regulated differential metabolite, and the green point represents a down-regulated differential metabolite. Gray represents a metabolite detected but not significantly different. Blue triangle marker points represent substances in terpenoid biosynthesis.

In the Y-tube olfactometer assays, compared with the blank control, male and female adults of *H. axyridis* had no significant preference for healthy *V. negundo* plants (female: *x* ^2 =^ 0.184, *P*=0.668; male: *x* ^2 =^ 1.14, *P*=0.286), but showed a significant preference for *V. negundo* previously infested by *A. gossypii* (female: *x* ^2 =^ 4.412, *P*=0.036; male: *x* ^2 =^ 7.407, *P*=0.006). Compared with healthy *V. negundo*, *H. axyridis* was more susceptible to plants previously infested by *A. gossypii* (female: *x* ^2 =^ 5.255, *P*=0.022; male: *x* ^2 =^ 8.395, *P*=0.004) (**Figure 3**; **Figure 4A, B**).

### Analysis of *V. negundo* plant volatile compounds

A projection to orthogonal partial least squares-discriminant analysis (OPLS-DA) using the contents of all detected volatiles showed a clear separation between herbivore-infested treatments and healthy plants. The first two significant OPLS components explained 47.6% and 32.9% of the total variance, respectively (**Figure 4C**). The results showed that the release of isogeraniol, 2-phenylethanol, 2,6-dimethyl-1,3,5,7-octatetraene, 2,3-dihydrobenzofuran and nonanal was significantly up-regulated, the release of 43 substances was significantly down-regulated, and the release of 41 substances was not significantly changed (**Figure 4D**, **Table 2**). Terpenoids are important components of herbivore induced plant volatiles. In the biosynthesis of terpenoids, it was found that the release of eucalyptol, α-terpineol, sclareol, (+)-Δ-cadinene and valencene decreased (**Figure S1**, **Table 2**).

**Table 2:** Five significantly up-regulated and five down-regulated substances in terpenoid biosynthesis.

Formula	Compounds	Class I	CAS	
C_10_H_18_O	Isogeraniol	Terpenoids	5944-20-7	up-regulated
C_8_H_10_O	2-Phenylethanol	Alcohol	60-12-8	up-regulated
C_10_H_14_	2,6-dimethyl-1,3,5,7-octatetraene	Terpenoids	460-01-5	up-regulated
C_8_H_8_O	2,3-Dihydrobenzofuran	Heterocyclic compound	496-16-2	up-regulated
C_9_H_18_O	Nonanal	Aldehyde	124-19-6	up-regulated
C_10_H_18_O	Eucalyptol	Terpenoids	470-82-6	down-regulated
C_10_H_18_O	α-Terpineol	Terpenoids	10482-56-1	down-regulated
C_15_H_24_	Valencene	Terpenoids	4630-07-3	down-regulated
C_15_H_24_	(+)-Δ-cadinene	Terpenoids	483-76-1	down-regulated
C_20_H_36_O_2_	Sclareol	Terpenoids	515-03-7	down-regulated

### Behavioral response of *Harmonia axyridis* adults to volatile odorants

**Figure 5:**
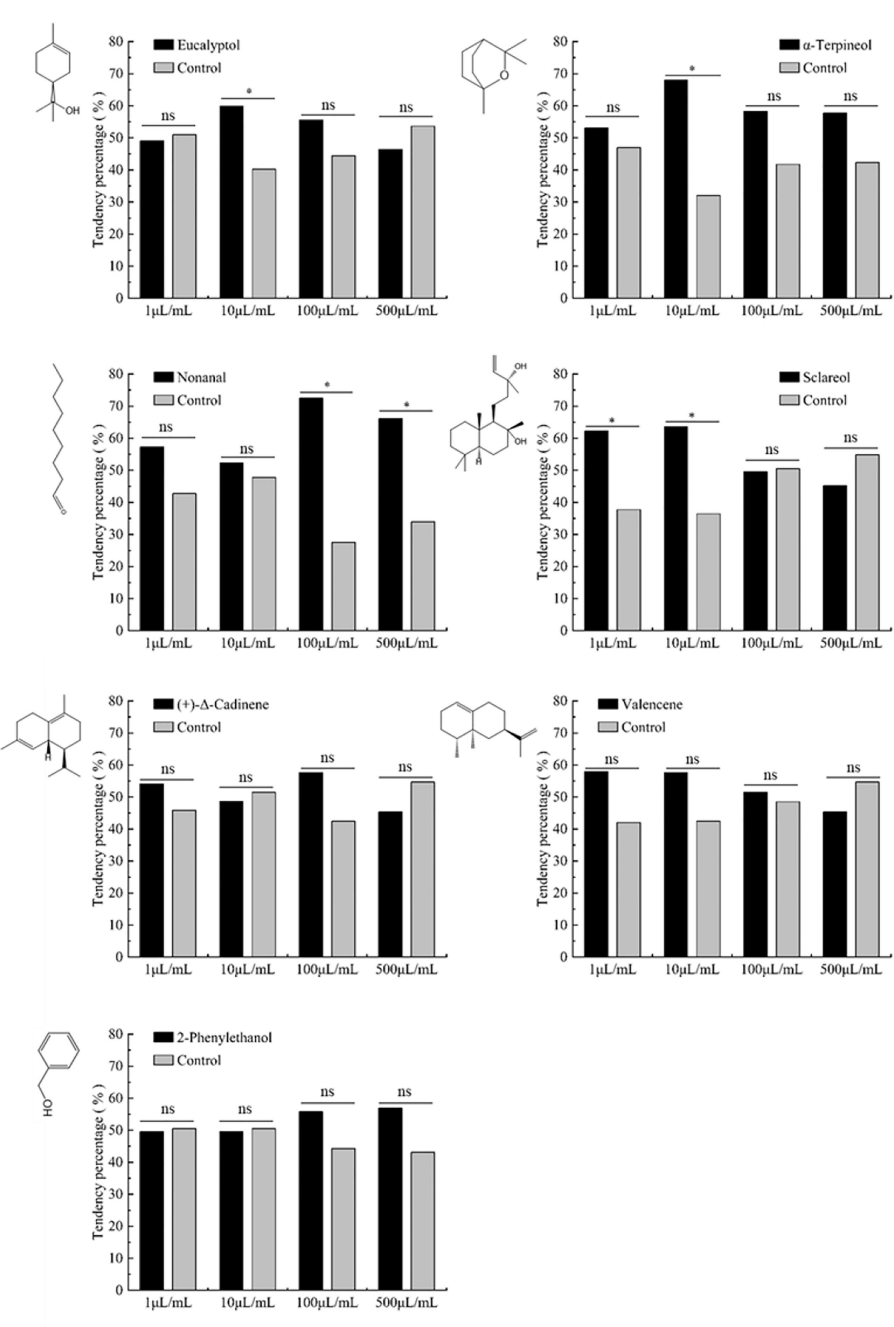
Behavioral responses of *H. axyridis* adults to key volatile compounds. Control for equal volume of paraffin liquid, Chi-square test was used between the test odor substances and the control group. “*” indicated that there was a significant difference in the preference of *H. axyridis* to the key odor substances and the control at the same concentration (*P*< 0.05) while “ns” indicated no significant difference.

The results of olfactory test showed that 10 μL/mL eucalyptol and α-terpineol significantly attracted *H. axyridis* (*x* ^2 =^ 4.121, *P*=0.042; *x* ^2 =^ 13.291, *P*<0.001, respectively). Low concentration (1 μL/mL and 10 μL/mL) of sclareol and high concentration (100 μL/mL and 500 μL/mL) of nonanal also significantly attracted *H. axyridis* (*x* ^2 =^ 6.377, *P*=0.012 and *x* ^2 =^ 7.042, *P*=0.008; *x* ^2 =^ 22.028, *P*<0.001 and *x* ^2 =^ 11.239, *P*<0.001, respectively). However, 2-phenylethanol, (+)-Δ-cadinene and valencene had no significant effect on *H. axyridis* attraction at different concentrations (**Figure 5**).

## Discussion

Non-crop habitat plants can play a role in attracting natural enemy insects, while the spill-over between the target area (e.g., crops and orchards) and non-crop habitat plants determines the effectiveness of pest control (Xie et al., 2012; Hatt et al., 2017; Xu et al., 2020). The most notable applications in crop protection are the push-pull strategies (Finch and Collier, 2000; Kleijn et al., 2011). Plant flower belt is one of the successful examples of push-pull strategy in agricultural production (Li et al., 2021). In greenhouse tomato production, *Carica papaya* L. (Caricaceae) can be used as a non-crop plant to breed *Encarsia sophia* Girault & Dodd (Hymenoptera: Aphelinidae) for the control of *Bemisia tabaci* Gennadius (Hemiptera: Aleyrodidae) (Xiao et al., 2011), while *Calendula officinalis* L. (Asteraceae) enhances the control of *Myzus persicae* Sulzer (Hemiptera: Aphididae) and *Frankliniella occidentalis* Pergande (Thysanoptera: Thripidae) by increasing the population density of *Orius sauteri* Poppius (Hemiptera: Anthocoridae) (Zhao et al., 2017) and reduces intraguild predation between predators and increases aphid biocontrol in tomato (Liang et al., 2022). In this experiment, we show that predatory ladybugs could migrate from *V. negundo* to peach orchards, and *V. negundo* release HIPVs to attract natural enemies when infected by herbivorous insects.

Plants change the characteristics of volatile organic compounds to cope with external stimuli. In response to herbivorous insect stimulation, HIPVs attract natural enemy insects (Robert et al., 2013). We showed that only the infection of *A. gossypii* can induce the attractions of *V. negundo* plant to the predator *H. axyridis*, indicating the importance of HIPVs to natural enemies in field. Moreover, the difference in content and diversity of HIPVs would account for the attractions of natural enemies (Heil, 2014; Turlings and Erb, 2018). In the present study, the release content of 2-phenylethanol and nonanal, the active ingredients attracting *H. axyridis*, increased after infested by *A. gossypii*. Structural diversity determines the complexity and diversity of plant volatile species and functions. Volatile substances with structural differences bind to specific olfactory proteins and exhibit different effects (Liu et al., 2020). Insects use a certain class of chemical information substances to achieve intraspecific information exchange (Nesbitt et al., 1979). Differences in functional groups lead to diverse chemical pheromone structures, resulting in more accurate and efficient information exchange or transfer (Liu et al., 2020). The substances that repel *M. persicae* have conjugated olefinic chemical structures (Liu et al., 2013). Diverse biological functions can also be achieved by changing the length, position and spatial structure of carbon chains or double bonds (Conte et al., 1990). *Cydia pomonella* Linnaeus (Lepidoptera: Tortricidae) information substance is two double-bond of 12 carbon linear alcohol (Yang et al., 2004). The five terpenoids in this study are all composed of isoprene as the basic carbon skeleton unit. Among them, eucalyptol, α-terpineol and sclareol can directly attract *H. axyridis*, while (+)-Δ-cadinene and valencene have no significant effect on *H. axyridis*. Compared with the previous three substances, they all contain two carbon-carbon double-bonds in molecular structure. Valencene can be used as an alternative component for mosquito control (Tisgratog et al., 2018) and valencene (0.3%) showed strong repellent properties to *Tribolium castaneum* Herbst (Coleoptera: Tenebrionidae) (Guo et al., 2019), so terpenoids may exert different functions by increasing or decreasing the number of double bonds. In addition, different functional groups show different olfactory perceptions. Nonanal contains only one main carbon chain structure and only one aldehyde functional group, which may lead to its significant attraction to *H. axyridis* at high concentrations.

The variety of plant volatiles complicates the analysis of their function. The formulations composed of MeSA and benzaldehyde can attract *Trichogramma dendrolimi* Matsumura (Hymenoptera: Trichogrammatidae), and other natural enemies (Zhao et al., 2022). Mixtures of β-pinene and limonene significantly increased the abundance of natural enemies such as *Coccinella septempunctat*a Linnaeus and *H. axyridis* (Wu et al., 2022). We found that low concentration of eucalyptol, α-terpineol and sclareol significantly attracted *H. axyridis* adults. This may be related to the reduced release of these compounds, although the effect of sclareol on natural enemy insects has rarely been reported to our knowledge. *H. axyridis* showed a significant preference for nonanal at high concentrations, which may be caused by the significant up-regulation of its release in *V. negundo*. 2-phenylethanol, (+)-Δ-cadinene and valencene have no effect on the behavior of *H. axyridis*, possibly because of its direct effect on pests or by altering plant resistance. Herbivore induced plant volatiles may only act on natural enemies or pests, and may also affect both simultaneously. For example (E)-β-Farnesene can repel aphids while attracting natural enemies (Beale et al., 2006; Xu et al., 2018b). Nonanal elicits electroantennogram response in female *Grapholita molesta* Busck (Lepidoptera: Tortricidae) (Xiang et al., 2017), and prevent *Ostrinia furnacalis* Guenée (Lepidoptera: Crambidae) from laying eggs on maize plants (Yu et al., 2020). However, in practical applications, the effect of volatile mixtures is often higher. A mixture of nonanal and (Z)-3-hexen-1-ol can significantly attract the syrphid fly *Paragus quadrifasciatus* Meigen (Diptera: Syrphidae) in the field (Yu et al., 2008), and the mixture of α-terpineole and 1,8-cineole repelled the fall armyworm *Spodoptera frugiperda* J. E. Smith (Lepidoptera: Noctuidae) (Lima et al., 2009). In recent years, a growing body of literature has shown that volatiles of plant can attract natural enemies or/and repel pests in pest management programs (Yao et al., 2021; Wang et al., 2022). That is, it is strategic to study the active substances that have attractive effects on *H. axyridis* and explore their mixed ratios for conservation biological control.

Impact of herbivory on HIPVs may depend on herbivorous insect species, density, and infection time, and external stimuli would also include soil chemical composition and temperature (Degenhardt et al., 2009; Cai et al., 2014; McCormick, 2016). For example, silicon affects HIPVs by regulating the jasmonic acid pathway, altering the mixed components of pest-induced volatiles released by rice after damage by *Capaphalocrocis medinalis* Guenée (Lepidoptera: Crambidae), and increasing attractiveness to parasitoids (Reynolds et al., 2016; Liu et al., 2017).

Genes change the release of volatiles, affecting the attractiveness of natural enemy insects (Xiao et al., 2012). Research on the regulatory genes of volatiles is a direct and effective method to verify the difference in release (Bruce et al., 2015). In addition, the behavioral responses of insects are regulated by external chemical signals, which are often identified by olfaction (Gadenne et al., 2016). Olfactory proteins in insects are very rich, which can specifically bind chemical information substances, and can also use an olfactory protein to perceive multiple signals, identify chemical signals and transmit information (Fan et al., 2011). There is a high matching specificity between queen pheromone 9-oxo-2-decenoic acid (9-ODA) and drone antennal olfactory protein *OR*11 (Wanner et al., 2007), *HoblCSP*1 and *HoblCSP*2 bind to odorants such as cinnamaldehyde (Sun et al., 2014), *HaxyOBP*13 and *HaxyOBP*14 had the highest expression in antennae and *HaxyOBP*5 could bind to methyl salicylate, nonanal and other substances (Qu et al., 2021; Qu et al., 2022). Therefore, we hypothesize that the substances with reduced release in the terpenoid biosynthetic pathway may also be key components to attract *H. axyridis*, that is, eucalyptol, α-terpineol, sclareol, (+)-Δ-cadinene and valencene may have attraction to *H.axyridis* or enhance its attractiveness. Exploring the genes regulating the synthesis and release of volatiles, analyzing the olfactory proteins involved in volatile binding and the odorant receptors involved in volatile recognition are helpful to regulate the ecological control of pests by chemical ecology methods.

Studies had showed that combining attractive synthetically produced HIPVs with functional plants which provide alternative resources to the targeted natural enemies can attract and retain efficient natural enemies in crop fields (Simpson et al., 2011; Jaworski et al., 2019). Lots of flowers play an important role in increasing fitness in predatory natural enemies (Wang et al., 2020; Fang et al., 2022). Flowers of *Perilla frutescens*, mixed with prey, have a positive effect on *H. axyridis* survival and early reproduction (Hatt and Osawa, 2019). *Vitex negundo* can provide sufficient high-quality nectar sources for bees during flowering (Su et al., 2000). And we found the visit of *H. axyridis* on *V. negundo* flowers in field. Future research could assess the fitness of nectar supporting the populations of natural enemies and evaluate the effect of *V. negundo* plantings on pest suppression in a diversity of adjacent crops. Thus, a “push and pull (i.e., nonanal)” or “attract (i.e., nonanal) and reward (i.e., *V. negundo* flowers)” strategy is a promising ecological practice to enhance conservation biological control in orchard.

## Data availability statement

The raw data supporting the conclusions of this article will be made available by the authors, without undue reservation.

## Author contributions

QX, CW, SW and XG conceived and designed research. QX, CW, DX, ZJ, and CZ conducted experiments and analyzed data. QX, CW and SH wrote and revised the paper. All authors contributed to the article and approved the submitted version.
