# Supplementary material for: Ecological function of key volatiles in Vitex negundo infested by Aphis gossypii
Source: Front Plant Sci. 2023 Jan 12;13:1090559. doi: 10.3389/fpls.2022.1090559 (PMC9879570; doi:10.3389/fpls.2022.1090559)
Supplement: Supplementary file 1 [file Image_1.pdf]

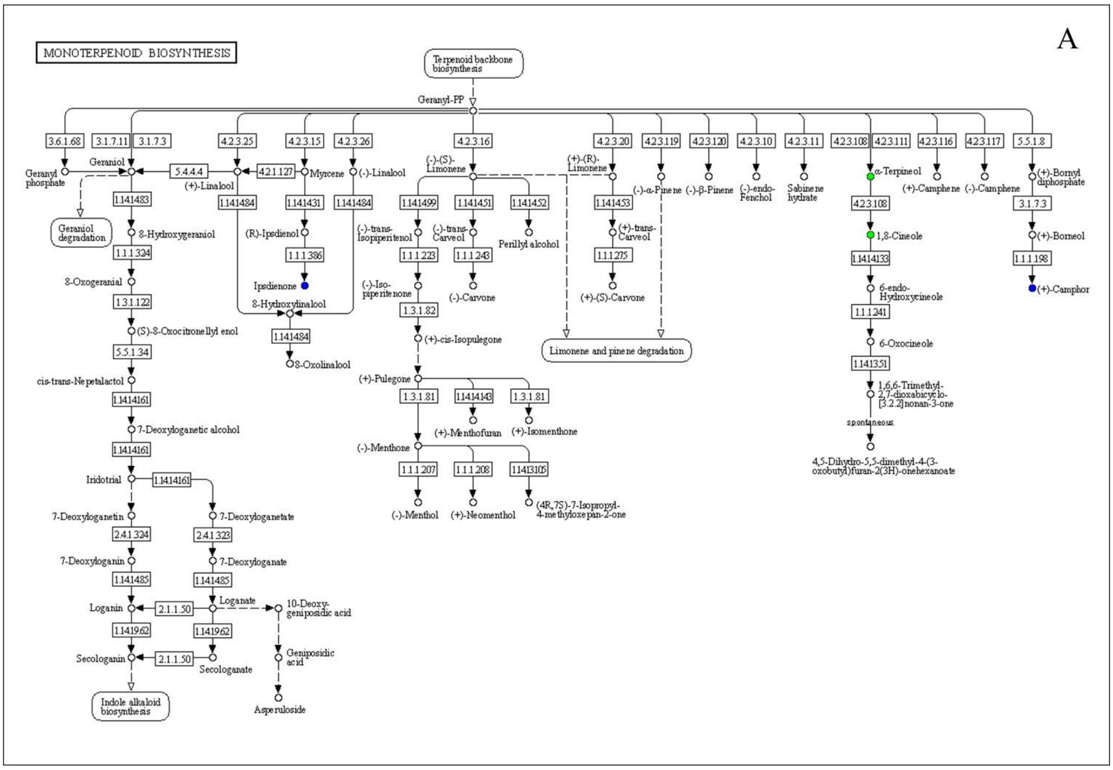
MONOTERPENOID BIOSYNTHESIS
A
Terpenoid backbone biosynthesis
Geranyl-PP
Geranyl phosphate
Geraniol
(+)-Linalool
Myrcene
(-)-Linalool
(-)-(S)-Limonene
(+)-(R)-Limonene
(-)-α-Pinene
(-)-β-Pinene
(-)-endo-Fenchol
Sabinene hydrate
α-Terpineol
(+)-Camphene
(-)-Camphene
(+)-Bornyl diphosphate
Geraniol degradation
8-Hydroxygeraniol
(R)-Ipsdienol
Ipsdienone
8-Oxogeranial
8-Hydroxylinalool
8-Oxolinalool
(S)-8-Oxocitronellyl enol
cis-trans-Nepetalactol
7-Deoxyloganetic alcohol
Iridotrial
7-Deoxyloganetin
7-Deoxyloganetate
7-Deoxyloganin
7-Deoxyloganate
Loganin
Loganate
10-Deoxygeniposidic acid
Geniposidic acid
Asperuloside
Secologanin
Secologanate
Indole alkaloid biosynthesis
(-)-trans-Isopiperitenol
(-)-trans-Carveol
Perillyl alcohol
(+)-trans-Carveol
(-)-Isopiperitenone
(-)-Carvone
(+)-(S)-Carvone
(+)-cis-Isopulegone
Limonene and pinene degradation
(+)-Pulegone
(+)-Menthofuran
(+)-Isomenthone
(-)-Menthone
(-)-Menthol
(+)-Neomenthol
(4R,7S)-7-Isopropyl-4-methyloxepan-2-one
1,8-Cineole
6-endo-Hydroxycineole
6-Oxocineole
1,6,6-Trimethyl-2,7-dioxabicyclo-[3.2.2]nonan-3-one
spontaneous
4,5-Dihydro-5,5-dimethyl-4-(3-oxobutyl)furan-2(3H)-onehexanoate
(+)-Borneol
(+)-Camphor

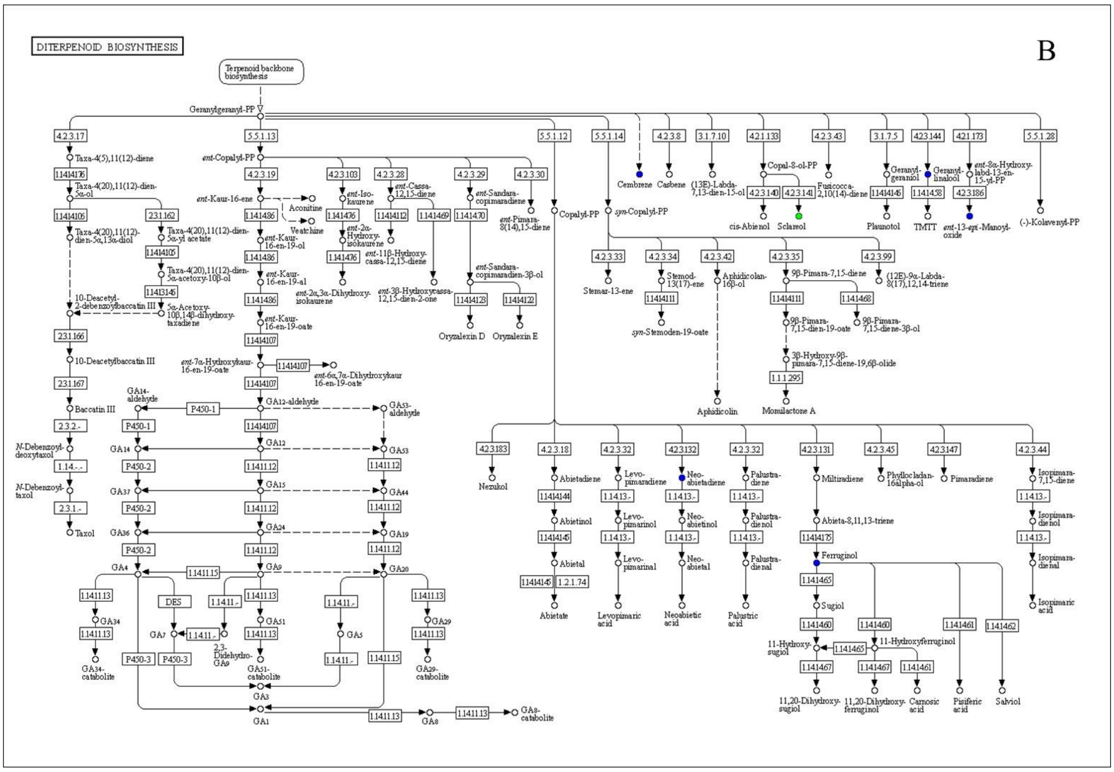
DITERPENOID BIOSYNTHESIS
B
Terpenoid backbone biosynthesis
Geranylgeranyl-PP
Taxa-4(5),11(12)-diene
ent-Copalyl-PP
Copalyl-PP
syn-Copalyl-PP
Cembrene
Casbene
Copal-8-ol-PP
Fusicocca-2,10(14)-diene
cis-Abienol
Sclareol
Plaunotol
TMTT
(-)-Kolavenyl-PP
ent-Kaur-16-ene
Aconitine
Veatchine
ent-Kaur-16-en-19-ol
ent-Kaur-16-en-19-al
ent-Kaur-16-en-19-oate
ent-7α-Hydroxykaur-16-en-19-oate
ent-6α,7α-Dihydroxykaur-16-en-19-oate
GA12-aldehyde
GA14-aldehyde
GA53-aldehyde
GA12
GA14
GA53
GA15
GA37
GA44
GA24
GA36
GA19
GA9
GA4
GA20
GA34
GA7
GA51
GA5
GA29
GA34-catabolite
GA51-catabolite
GA29-catabolite
GA3
GA1
GA8
GA8-catabolite
10-Deacetylbaccatin III
Baccatin III
N-Debenzoyl-deoxytaxol
N-Debenzoyl-taxol
Taxol
Oryzalexin D
Oryzalexin E
Stemar-13-ene
syn-Stemoden-19-oate
Aphidicolin
Momilactone A
Nezukol
Abietadiene
Abietinol
Abietal
Abietate
Levopimaradiene
Levopimarinol
Levopimaral
Levopimaric acid
Neoabietadiene
Neoabietinol
Neoabietal
Neoabietic acid
Palustradiene
Palustradienol
Palustradienal
Palustric acid
Miltiradiene
Abieta-8,11,13-triene
Ferruginol
Sugiol
11-Hydroxyferruginol
11-Hydroxysugiol
11,20-Dihydroxysugiol
11,20-Dihydroxyferruginol
Carnosic acid
Pisiferic acid
Salviol
Phyllocladan-16alpha-ol
Pimaradiene
Isopimara-7,15-diene
Isopimaradienol
Isopimaradienal
Isopimaric acid

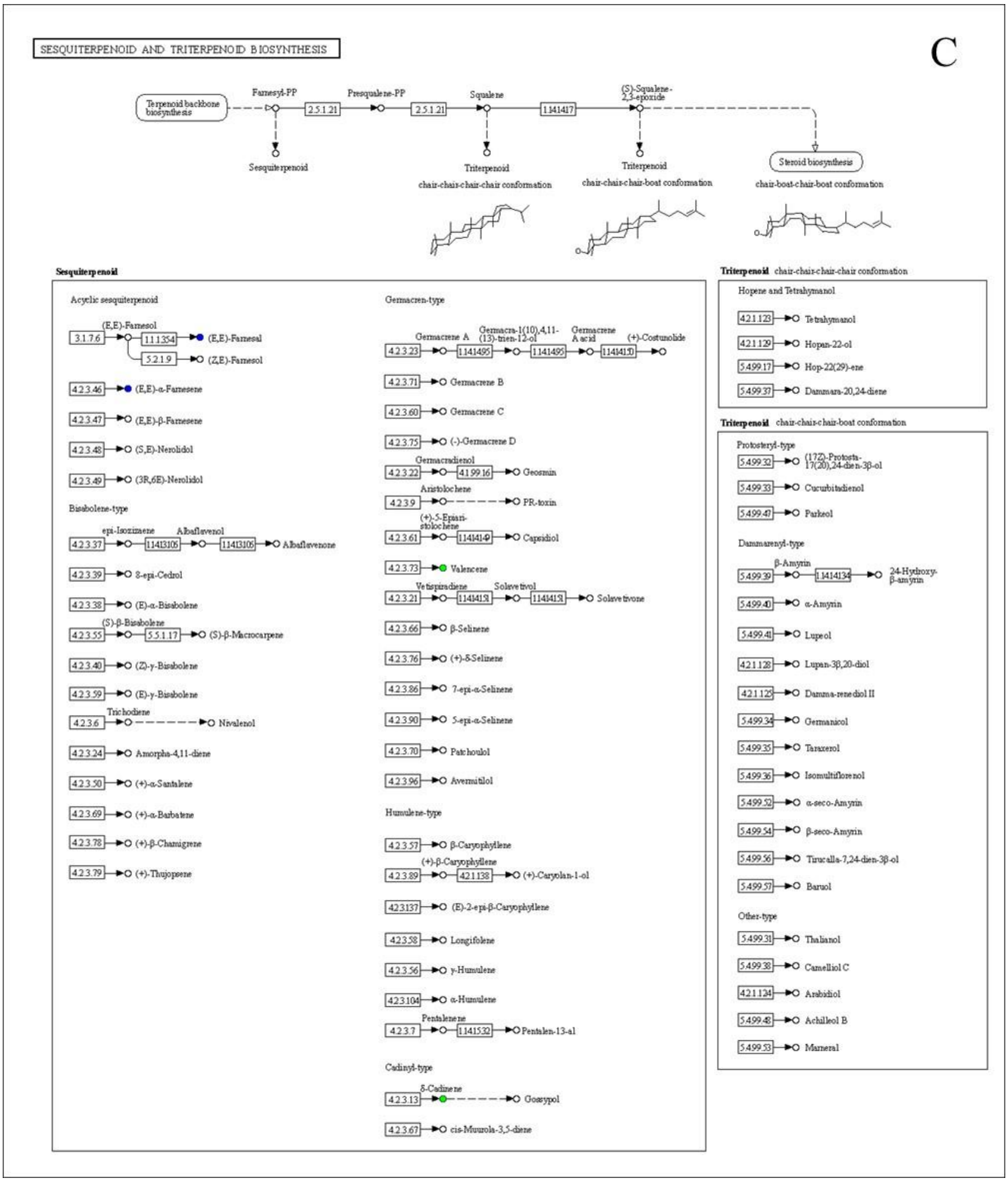

FIGURE S1 | The positions of key volatile compounds in monoterpenoid biosynthesis (A), diterpenoid biosynthesis (B) and sesquiterpenoid and triterpenoid biosynthesis (C)

Note: Green indicates that the metabolite content was significantly down-regulated in the experimental group, blue indicates that the metabolite was detected but did not change significantly.
